# Water Quality as a Predictor of *Legionella* Positivity of Building Water Systems

**DOI:** 10.3390/pathogens8040295

**Published:** 2019-12-13

**Authors:** David Pierre, Julianne L. Baron, Xiao Ma, Frank P. Sidari, Marilyn M. Wagener, Janet E. Stout

**Affiliations:** 1 Special Pathogens Laboratory, Pittsburgh, PA 15219, USA; dpierre@specialpathogenslab.com (D.P.); jbaron@specialpathogenslab.com (J.L.B.); maxiaosean1219@gmail.com (X.M.);; 2 School of Medicine, University of Pittsburgh, Pittsburgh, PA 15261, USA; mmw5@pitt.edu; 3 Department of Civil and Environmental Engineering, University of Pittsburgh, Pittsburgh, PA 15261, USA

**Keywords:** *Legionella*, distal site positivity, hot water return line, chlorine, HPC, temperature, water management

## Abstract

Testing drinking water systems for the presence of *Legionella* colonization is a proactive approach to assess and reduce the risk of Legionnaires’ disease. Previous studies suggest that there may be a link between *Legionella* positivity in the hot water return line or certain water quality parameters (temperature, free chlorine residual, etc.) with distal site *Legionella* positivity. It has been suggested that these measurements could be used as a surrogate for testing for *Legionella* in building water systems. We evaluated the relationship between hot water return line *Legionella* positivity and other water quality parameters and *Legionella* colonization in premise plumbing systems by testing 269 samples from domestic cold and hot water samples in 28 buildings. The hot water return line *Legionella* positivity and distal site positivity only demonstrated a 77.8% concordance rate. Hot water return line *Legionella* positivity compared to distal site positivity had a sensitivity of 55% and a specificity of 96%. There was poor correlation and a low positive predictive value between the hot water return line and distal outlet positivity. There was no correlation between *Legionella* distal site positivity and total bacteria (heterotrophic plate count), pH, free chlorine, calcium, magnesium, zinc, manganese, copper, temperature, total organic carbon, or incoming cold-water chlorine concentration. These findings suggest that hot water return line *Legionella* positivity and other water quality parameters are not predictive of distal site positivity and should not be used alone to determine the building’s *Legionella* colonization rate and effectiveness of water management programs.

## 1. Introduction

*Legionella* is considered an opportunistic human pathogen and these bacteria have been found in up to 70% of building water systems [1]. In recent molecular studies, *Legionella pneumophila* was isolated from 38% of buildings sampled including 42% of residences and 35% of office buildings [2], and in 47% of all taps in a study of buildings and homes in the United States [3]. *Legionella* colonization of potable water systems can pose a public health risk, especially for immunocompromised individuals [1,4].

Several organizations including ASHRAE (formerly known as the American Society of Heating, Refrigerating and Air-Conditioning Engineers), the World Health Organization, the American Industrial Hygiene Association, and the Centers for Disease Control and Prevention recommend the creation of water management programs aimed at preventing the growth and spread of *Legionella* and other waterborne pathogens [5,6,7,8].

Testing water for the presence of *Legionella* is the most direct means of determining whether the building water system is colonized by *Legionella* [9,10] and its usefulness has been discussed in multiple technical guidelines [7,11,12]. The correlation with disease risk has been well established in healthcare facilities [13,14,15,16], but risk has also been demonstrated in hotels and other commercial properties. Rather than recommend testing for the bacteria, some guidelines and standards have suggested that building design or physical and chemical properties of the water can be used as predictors of risk or to demonstrate that water management programs have effectively controlled the growth and spread of *Legionella* [5,8].

For example, ASHRAE selected certain physical properties of buildings as requisite characteristics for requiring a water management program [5]. This included building height (greater than 10 stories including below grade), which had previously been found to have an increased presence of *Legionella* in the buildings’ water heaters [17,18]. The Centers for Disease Control and Prevention (CDC) and the Centers for Medicare and Medicaid Services (CMS) recommend that temperature, pH, and free chlorine be tested at numerous outlets when doing a *Legionella* risk assessment [8,19]. Others have suggested that the temperature of the hot water in the recirculation line of a building [20] or other water quality parameters could predict the presence or absence of *Legionella* at the distal outlets (faucets and showers) [20,21,22,23].

There is a problem with these recommendations. There is little data to support them. If such monitoring is to be performed and relied upon as part of risk assessments and water management programs, the expectation is that this information will have some relationship to either the presence or absence of *Legionella*.

It is important that we better understand these assumptions. Therefore, we performed a large-scale field investigation to evaluate the presence of *Legionella* in premise plumbing systems in 28 buildings in New York City, San Francisco, and New Jersey. The objective of the study was to (1) evaluate the potential of using hot water return line *Legionella* positivity as an indicator of distal site *Legionella* colonization risk in these buildings and (2) evaluate the correlation between water quality parameters and the presence of *Legionella* in water systems.

## 2. Results

### 2.1. Legionella Positivity Correlation

**Figure 1:**
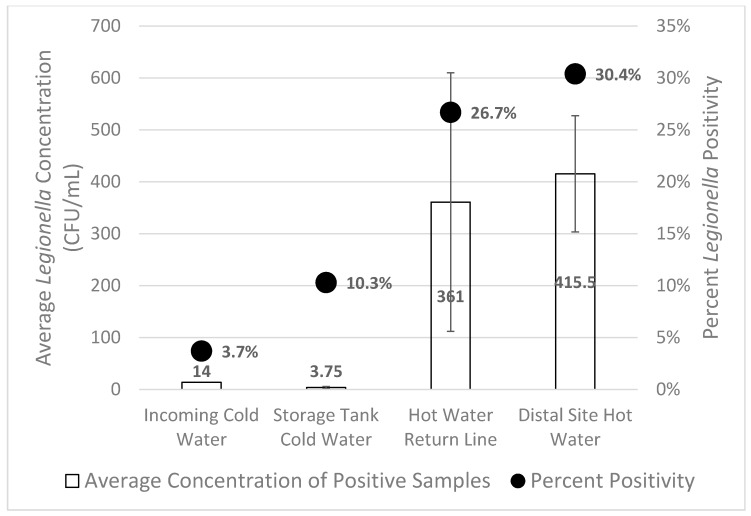
Percent *Legionella* positivity and concentration was highest in distal site hot water and hot water return lines. The bars represent the standard error of the mean for the average *Legionella* concentration.

A total of 269 samples were cultured for *Legionella* from domestic cold and hot water samples in 28 different buildings and from 45 recirculating hot water systems. *Legionella* was cultured from 65/269 (24.2%) samples from 15/28 (53.6%) buildings sampled. The hot water return line sample was positive in 12/45 (26.7%) systems (Figure 1). Positive distal sites (faucets) were observed in 20/45 (44.4%) of the hot water systems. *L*. *pneumophila* was the only species of *Legionella* isolated from these water samples. There was a trend towards larger buildings having more distal site positivity, however this was not statistically significant (*p* = 0.06).

**Figure 2:**
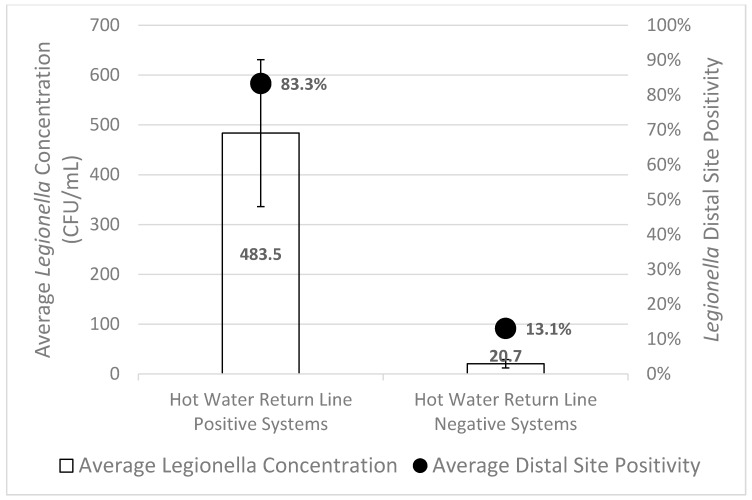
*Legionella* distal site positivity and concentration was highest for hot water systems with *Legionella* positive hot water return lines. The bars represent the standard error of the mean for average *Legionella* concentration.

*Legionella* was isolated from at least one distal site in 91.7% (11/12) of the hot water systems that also had *Legionella* isolated from the hot water return line, with an average distal site positivity of 83.3% ± 8.7% (Figure 2). *Legionella* was isolated from at least one distal site in 27.3% (9/33) of the hot water systems with a *Legionella* negative hot water return line, with an average distal site positivity of 13.1% ± 4.3% (Figure 2). In 35 of the 45 sampled hot water systems, there was agreement between *Legionella* distal site positivity and hot water return line *Legionella* positivity, resulting in a 77.8% concordance rate.

We then analyzed if hot water return line *Legionella* positivity was able to correctly predict whether the distal sites would be positive or negative for *Legionella*. Hot water return line positivity was related to distal site positivity (*p* = 0.002), with a sensitivity of only 55% (11/20) and a specificity of 96% (24/25). However, when hot water return line positivity was used as a screening tool for distal site positivity, the positive predictive value was 91.7% and the negative predictive value was only 72.7%. The average distal site concentration of *Legionella* in systems with a positive hot water return was 483.5 ± 147.4 CFU/mL, versus 20.7 ± 8.4 CFU/mL in negative hot water returns (*p* < 0.003) (Figure 2).

### 2.2. Heterotrophic Plate Count (HPC) and Chemical Parameter Correlation

**Figure 3:**
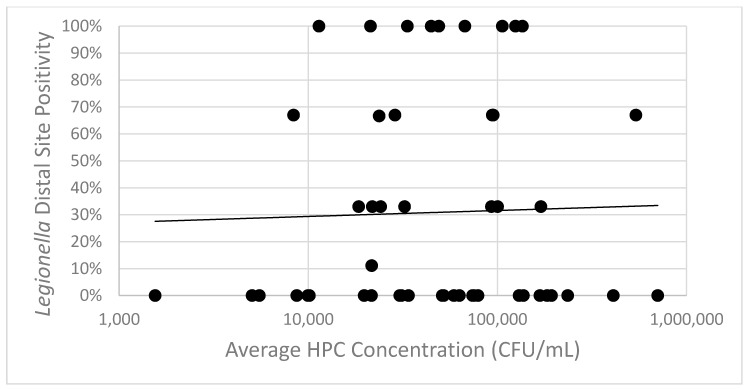
*Legionella* distal site positivity and average distal site HPC concentration are not significantly associated. Average HPC concentration is represented on a logarithmic scale x-axis. A line of best fit has been added to show the relationship between HPC concentration and distal site positivity.

All samples collected were also cultured for heterotrophic plate count (HPC) bacteria (Table 1). HPC concentrations in hot water samples ranged from 3 CFU/mL to 2,100,000 CFU/mL. Statistical analysis showed no correlation between distal site HPC concentration and *Legionella* distal site positivity (*p* = 0.788) (Figure 3). The best-fit linear regression line demonstrates that HPC concentration explains only 0.68% of the variance in *Legionella* distal site positivity (R^2^ = 0.0068).

The water from distal outlets was analyzed for pH, free chlorine, calcium (Ca), magnesium (Mg), zinc (Zn), manganese (Mn), copper (Cu), and TOC. These results were analyzed for correlation with distal site positivity (Table 1). None of the measured parameters were shown to have a correlation with *Legionella* distal site positivity (*p* values > 0.05). Hot water return line pH, free chlorine, calcium, magnesium, zinc, manganese, and copper also were not correlated with *Legionella* distal site positivity (*p* values > 0.05). No comparisons could be made between either distal site or hot water return line iron (Fe) or lead (Pb) concentrations and *Legionella* distal site positivity because the concentrations were below the lower detection limit of the test method in 49% and 77% of samples for iron and lead, respectively.

### 2.3. Temperature Correlation

Distal site temperatures averaged 119.95 °F after a 1-min flush. Distal site temperature was not statistically related to the distal site *Legionella* positivity (*p* = 0.170). Distal sites with no *Legionella* recovered trended towards higher hot water return temperatures, with an average 9.7 °F higher temperature. However, this was not statistically significant (*p* = 0.0687).

**Figure 4:**
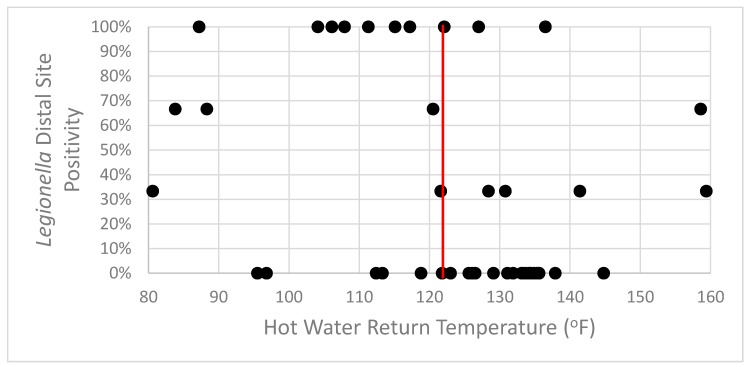
*Legionella* distal site positivity was seen in systems with hot water return line temperatures above and below the recommended threshold. The red vertical line represents the 124 °F threshold value.

When using 124 °F, the minimum recommended return circulation temperature [24], as a threshold for *Legionella* distal site positivity, there was an association between the two values (*p* = 0.013) (Figure 4). This hot water return temperature threshold value had a sensitivity of 65% (13/20) and a specificity of 72% (18/25) for determining the *Legionella* distal site positivity. However, the positive predictive value was only 65% (13/20) and the negative predictive value was 72% (18/25). This recommended temperature threshold is a poor screening test for distal site positivity (receiver operating characteristic (ROC) curve area = 0.68). In the buildings with a hot water return temperature > 124 °F, 28% (7/25) were still positive for *Legionella* in distal sites.

### 2.4. Incoming Cold-Water Chlorine Concentration

**Figure 5:**
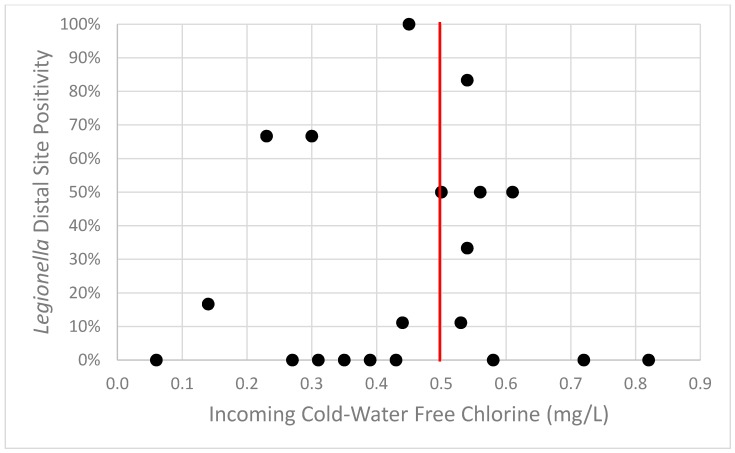
*Legionella* distal site positivity was seen in buildings with incoming cold-water free chlorine above and below the recommended threshold. The red vertical line represents the 0.5 mg/L threshold value.

Incoming cold-water chlorine concentration was analyzed to determine if the concentration could be used as a predictor of *Legionella* distal site positivity. Incoming cold-water chlorine measurements were available from 20 buildings. Using 0.5 mg/L as the threshold for the acceptable level of free residual chlorine found in drinking water [25], there was no correlation between incoming chlorine concentration and *Legionella* positivity (*p* = 0.582) (Figure 5). This 0.5 mg/L incoming chlorine threshold fails as a screening tool for *Legionella* distal site positivity (ROC curve area = 0.47).

## 3. Discussion

Public health agencies and some guidance documents recommend monitoring temperature and water quality parameters as part of *Legionella* risk assessments and water management programs. A reasonable assumption in following these recommendations is that there is some relationship to *Legionella* presence or absence.

However, few studies have been conducted to evaluate these relationships and to determine if any of these surrogate measurements can substitute for *Legionella* sampling in assessing risk or effectiveness of control measures [20,21,22,23] and there is a growing interest for a more efficient sampling approach for water system *Legionella* testing [20,26]. In this study we measured *Legionella* positivity, heterotrophic plate count bacteria, and other water physicochemical parameters, including temperature, free chlorine concentration, pH, calcium, magnesium, total organic carbon, iron, zinc, lead, manganese, and copper concentrations. We also evaluated the correlation between hot water return line *Legionella* positivity or these other water quality parameters to determine if any of these relationships were predictive of *Legionella* distal site positivity.

Our analysis showed that the concordance rate between hot water return line *Legionella* positivity and distal site *Legionella* positivity was 77.8%. This was similar to a previous report of 79% concordance between the hot water recirculation loop and distal sites [20]. In the current study, we further determined the sensitivity and specificity of using hot water return line *Legionella* positivity as a screening tool to predict distal site *Legionella* positivity. The low sensitivity (55%) indicates a low probability of finding distal site *Legionella* colonization based on the hot water return line *Legionella* positivity alone. In many cases, hot water return lines that yielded no *Legionella* had positive distal sites in that system.

Studies have linked the presence of *Legionella* in building water systems to water physicochemical parameters such as trace elements concentration, pH, and temperature. A significant association between *Legionella* presence and concentrations of Mn, Zn, and Fe was reported previously [22]. In another report, iron was significantly higher (average 1.43 mg/L) in *Legionella* positive public building water samples compared to *Legionella* negative samples (average 0.09 mg/L) [27]. This association was also seen with residential water systems [28]. In the present study, statistical analysis of the correlation between *Legionella* positivity and Fe as well as Pb concentration was not possible, because 49% and 77% of total samples had Fe and Pb concentrations lower than the lower detection limit (0.06 and 0.002 mg/L, respectively). Copper concentrations of > 0.05 mg/L have been associated with a lower risk of *Legionella* colonization [29].

We observed no statistically significant correlation between distal site *Legionella* positivity and HPC, temperature, pH, free chlorine (incoming cold water and distal site), Ca, Mg, Zn, Mn, Cu, and hot water return TOC. We found HPC concentration to be a poor predictor of *Legionella* positivity. HPC concentration was only able to explain 0.68% of the variation in *Legionella* distal site positivity. These results are consistent with a previous report that also showed the lack of correlation between total bacterial counts, measured by HPC, and *Legionella* colonization [23].

*Legionella* negative hot water systems trended towards higher temperature, Ca concentration, and lower hot water return TOC concentration, however, these were not statistically significant. In contrast to one other study, we found no correlation between *Legionella* colonization and manganese in building water systems [30]. *Legionella* positive systems trended towards higher Mn concentration on average, although this relationship was also not statistically significant.

From our experience, buildings > 10 stories high often have multiple centralized hot water systems installed to serve different building zones. The complexity of these centralized hot water systems lead to favorable environments for *Legionella* colonization, such as increased water age, favorable temperatures, and a lack of disinfectant residual [2,18,27,31,32]. Previous studies have shown a correlation between the building size and *Legionella* growth. These studies have indicated that larger buildings (>10 stories) and those with centralized hot water systems are more likely to support *Legionella* growth [17,18]. We did not see a statistical relationship in our study, between building size and *Legionella* positivity, however our data suggest the need for further investigation with larger data sets.

The risk of acquiring Legionnaires’ disease has been previously associated with high levels of distal site *Legionella* positivity (>30%) [1,9,13,14,15]. Localized *Legionella* colonization at the point-of-use such as faucets and shower heads had been frequently observed and linked to the risk of susceptible individuals [33,34,35] and would serve as a patient–water system interaction point. Monitoring a facility’s water system for *Legionella* colonization involves sampling distal hot water outlets regularly, which may necessitate the collection of numerous samples, especially for large facilities with multiple hot water systems [6,20].

Based on our study, sampling and culturing only the hot water return lines for *Legionella* presence demonstrates a low sensitivity of identifying *Legionella* colonization and therefore Legionnaires’ disease risk. Similarly, the measured water quality parameters were not predictors of *Legionella* distal site positivity. Hot water temperature or incoming cold-water chlorine thresholds, 124 °F and 0.5 mg/L, respectively, also did not serve as good screening tools for *Legionella* colonization. In facilities with high risk residents, such as hospitals and long-term care facilities, a more conservative approach of direct sampling of at least 10 distal sites is recommended [9,36,37]. Based on our results, we recommend that in lower risk facilities, such as commercial or administrative buildings, sampling at least three distal sites and the hot water return should be done for routine surveillance in each hot water system. If positive samples are found, a more thorough examination of the extent and location of colonization may be warranted especially in office buildings where *Legionella* has been found to persist regardless of building age [2]. In ASHRAE Standard 188, an important part of any water management program is to ensure that there is validation of the program’s efficacy. This is to ensure the water management program is controlling identified hazardous conditions, specifically the risk of *Legionella* growth and spread. Our results demonstrate that these surrogate measurements cannot be used to validate the control of *Legionella* risk at a facility because they are not predictive of the presence or absence of *Legionella* species.

## 4. Materials and Methods

### 4.1. Sample Collection and Onsite Water Quality Parameter Measurements

Bulk water samples were collected from 28 buildings in New York City (25 buildings), San Francisco (two buildings), and New Jersey (one building) from March to September 2015. The sampled buildings included commercial buildings ranging from 5 to 57 floors. Samples were collected from the incoming cold-water per building, cold-water storage tank per cold water system, three hot water distal outlets per hot water system (near, mid and far), and the hot water return line from each hot water system.

Cold water and hot water return line samples were collected after a 1-min flush. A 1 L sample with sodium thiosulfate for microbiological analyses and a 250 mL sample preserved with nitric acid for metal analyses (iron (Fe), copper (Cu), lead (Pb), zinc (Zn), calcium (Ca), magnesium (Mg), and manganese (Mn)) were collected. Hot water distal outlet samples were collected and treated as above, but the 1 L microbiological sample was taken prior to flushing. Additionally, for hot water return line samples, two 50 mL vials with hydrochloric acid preservative were collected for total organic carbon (TOC) testing. Measurements for temperature, pH, and free chlorine were conducted onsite after sample collection.

Temperature, pH, and free chlorine residual concentration were measured on-site at the time of sample collection using a digital thermometer, portable Hach 900 colorimeter, and Oakton Acorn pH meter following the manufacturer’s protocols. Samples for microbiological analyses were shipped on ice overnight to Special Pathogens Laboratory (Pittsburgh, PA, USA) and samples for metal analyses and total organic carbon testing were shipped to ALS Environmental (Middletown, PA, USA).

### 4.2. Microbiological Analyses

*Legionella* culture was conducted using buffered charcoal yeast extract agar (BCYE) (Remel, Lenexa, KS, USA) and selective media supplemented with glycine, vancomycin, and polymyxin B (DGVP) [38] using a modified ISO method [39,40]. Heterotrophic plate count (HPC) bacteria culture was performed using R2A agar (Remel, Lenexa, KS, USA) following standard method 9215B [41]. Culture media plates were prepared in-house with dehydrated media as noted above.

### 4.3. Statistical Analyses

For categorical data (building size, dichotomized threshold variables) *Legionella* positivity was compared using the Chi square test. Logistic models were used to evaluate *Legionella* positivity (presence/absence) and continuous variables (HPC, temperature, pH, free chlorine, calcium, magnesium, zinc, manganese, copper, and total organic carbon). A receiver operating characteristic (ROC) curve and the area under the curve (AUC) were generated to evaluate the utility of hot water return line temperature (dichotomized by recommended threshold) for predicting *Legionella* positivity. All statistics were performed using Stata version 13.0 (Stata Corp, College Station, TX, USA). A linear regression, and resulting R^2^ value, was generated to evaluate the utility of HPC concentration for predicting *Legionella* positivity using Microsoft Excel.

## 5. Conclusions

Water quality measurements, including hot water return line *Legionella* positivity, total bacterial counts, temperature, and other physicochemical parameters, have previously been sought or suggested as alternative approaches to determine the *Legionella* risk for a building’s water system instead of directly culturing the system. We found a concordance rate of only 77.8% between hot water return *Legionella* positivity and distal site *Legionella* positivity. Additionally, using hot water return line positivity as a predictor for *Legionella* distal site positivity had a sensitivity of only 55% and a specificity of 96%. There was no significant correlation between *Legionella* positivity and any water quality parameter (HPC, temperature, incoming cold-water chlorine, or physicochemical concentrations) tested. Neither hot water return line *Legionella* positivity nor other water quality parameters are suitable as a surrogate or stand-alone replacement for sampling and culturing distal sites for *Legionella* colonization in building water systems, especially in facilities with higher-risk populations.

## Figures and Tables

**Table 1:** Average concentrations (±standard deviation) of total bacteria (heterotrophic plate count—HPC) and physicochemical parameters.

	Incoming Cold Water	Storage Tank Cold Water	Hot Water Return	Distal Site Hot Water
HPC (CFU/mL)	18,954 ± 71,478.3	7254 ± 20,138.3	11,360.4 ± 25,094.9	92,125.4 ± 221,547.2
Temperature (°F, 30 s flush)	NT	NT	NT	109.36 ± 21.85
Temperature (°F, 1 min flush)	63.12 ± 9.22	62.66 ± 9.76	122.80 ± 17.85	119.95 ± 73.22
pH	7.29 ± 0.78	7.17 ± 0.69	6.97 ± 0.67	6.93 ± 0.63
Free Cl (mg/L)	0.34 ± 0.24	0.23 ± 0.16	0.07 ± 0.13	0.05 ± 0.09
Fe (mg/L)	NA	NA	NA	NA
Ca (mg/L)	7.16 ± 4.41	6.56 ± 1.58	6.39 ± 1.19	6.61 ± 3.09
Mg (mg/L)	1.66 ± 1.09	1.46 ± 0.46	1.72 ± 1.80	1.79 ± 1.81
Zn (mg/L)	0.03 ± 0.04	0.02 ± 0.03	0.05 ± 0.09	0.04 ± 0.06
Pb (mg/L)	NA	NA	NA	NA
Mn (mg/L)	0.02 ± 0.03	0.02 ± 0.02	0.03 ± 0.12	0.03 ± 0.11
Cu (mg/L)	0.09 ± 0.20	0.05 ± 0.06	0.30 ± 0.51	0.19 ± 0.23
TOC (mg/L)	NT	NT	1.85 ± 0.26	NT

NA: 48.7% samples were with Fe concentration below detection limit of 0.03 mg/L, detectable Fe concentration ranged from 0.03 to 4.6 mg/L; 76.2% samples were with Pb concentration below detection limit of 0.001 mg/L; Pb concentrations in detectable samples ranged from 0.001 to 0.63 mg/L. NT: not tested. TOC = Total Organic Carbon.

## References

[1] Stout J.E., Yu V.L., Muraca P., Joly J., Troup N., Tompkins L.S. (1992). Potable Water as a Cause of Sporadic Cases of Community-Acquired Legionnaires’ Disease. N. Engl. J. Med..

[2] Donohue M.J., King D., Pfaller S., Mistry J.H. (2019). The sporadic nature of Legionella pneumophila, Legionella pneumophila Sg1 and Mycobacterium avium occurrence within residences and office buildings across 36 states in the United States. J. Appl. Microbiol..

[3] Donohue M.J., O’Connell K., Vesper S.J., Mistry J.H., King D., Kostich M., Pfaller S. (2014). Widespread Molecular Detection of Legionella pneumophila Serogroup 1 in Cold Water Taps across the United States. Environ. Sci. Technol..

[4] Anaissie E.J., Penzak S.R., Dignani M.C. (2002). The hospital water supply as a source of nosocomial infections: A plea for action. Arch. Intern. Med..

[5] ASHRAE (2018). ANSI/ASHRAE Standard 188-2018. Legionellosis: Risk Management for Building Water Systems.

[6] World Health Organization (WHO) (2007). Legionella and the Prevention of Legionellosis.

[7] AIHA (2015). Recognition, Evaluation, and Control of Legionella in Building Water Systems.

[8] Centers for Disease Control and Prevention (CDC) (2017). Developing a Water Management Program to Reduce Legionella Growth & Spread in Buildings—A Practical Guide to Implementing Industry Standards.

[9] Stout J.E., Yu V.L. (2010). Environmental culturing for Legionella: Can we build a better mouse trap?. Am. J. Infect. Control.

[10] Stout J.E., Muder R.R., Mietzner S., Wagener M.M., Perri M.B., DeRoos K., Goodrich D., Arnold W., Williamson T., Ruark O. (2007). Role of environmental surveillance in determining the risk of hospital-acquired legionellosis: A national surveillance study with clinical correlations. Infect. Control Hosp. Epidemiol. Off. J. Soc. Hosp. Epidemiol. Am..

[11] Kozak N.A., Lucas C.E., Winchell J.M., Buchrieser C., Hilbi H. (2013). Identification of Legionella in the Environment. Legionella: Methods and Protocols.

[12] ASTM (2015). D5952-08(2015), Standard Guide for the Inspection of Water Systems for Legionella and the Investigation of Possible Outbreaks of Legionellosis (Legionnaires’ Disease or Pontiac Fever).

[13] Best M., Yu V.L., Stout J., Goetz A., Muder R.R., Taylor F. (1983). Legionellaceae in the hospital water supply--epidemiological link with disease and evaluation of a method of control of nosocomial Legionnaires’ disease and Pittsburgh pneumonia. Lancet.

[14] Kool J.L., Bergmire-Sweat D., Butler J.C. (1999). Hospital characteristics associated with colonization of water systems by Legionella and risk of nosocomial legionnaires’ disease: A cohort study of 15 hospitals. Infect. Control Hosp. Epidemiol..

[15] Sabria M., Modol J.M., Garcia-Nunez M., Reynaga E., Pedro-Botet M.L., Sopena N. (2004). Environmental cultures and hospital-acquired Legionnaires’ disease: A 5-year prospective study in 20 hospitals in Catalonia, Spain. Infect. Control Hosp. Epidemiol. Off. J. Soc. Hosp. Epidemiol. Am..

[16] Boccia S., Laurenti P., Borella P., Moscato U., Capalbo G., Cambieri A., Amore R., Quaranta G., Boninti F., Orsini M. (2006). Prospective 3-year surveillance for nosocomial and environmental Legionella pneumophila: Implications for infection control. Infect. Control Hosp. Epidemiol. Off. J. Soc. Hosp. Epidemiol. Am..

[17] Flannery B., Gelling L.B., Vugia D.J., Weintraub J.M., Salerno J.J., Conroy M.J., Stevens V.A., Rose C.E., Moore M.R., Fields B.S. (2006). Reducing Legionella colonization in water systems with monochloramine. Emerg. Infect. Dis..

[18] Borella P., Montagna M.T., Romano-Spica V., Stampi S., Stancanelli G., Triassi M., Neglia R., Marchesi I., Fantuzzi G., Tato D. (2004). Legionella infection risk from domestic hot water. Emerg. Infect. Dis..

[19] Wright D.R., Centers for Medicare & Medicaid Services (2017). Requirement to Reduce Legionella Risk in Healthcare Facility Water Systems to Prevent Cases and Outbreaks of Legionnaires’ Disease (LD). S&C 17-30-Hospitals/CAHs/NHs.

[20] Ditommaso S., Giacomuzzi M., Gentile M., Moiraghi A.R., Zotti C.M. (2010). Effective environmental sampling strategies for monitoring *Legionella* spp contamination in hot water systems. Am. J. Infect. Control.

[21] Bédard E., Fey S., Charron D., Lalancette C., Cantin P., Dolcé P., Laferrière C., Déziel E., Prévost M. (2015). Temperature diagnostic to identify high risk areas and optimize Legionella pneumophila surveillance in hot water distribution systems. Water Res..

[22] Bargellini A., Marchesi I., Righi E., Ferrari A., Cencetti S., Borella P., Rovesti S. (2011). Parameters predictive of Legionella contamination in hot water systems: Association with trace elements and heterotrophic plate counts. Water Res..

[23] Duda S., Baron J.L., Wagener M.M., Vidic R.D., Stout J.E. (2015). Lack of correlation between Legionella colonization and microbial population quantification using heterotrophic plate count and adenosine triphosphate bioluminescence measurement. Environ. Monit. Assess..

[24] ASHRAE (2000). ASHRAE Guideline 12-2000. Minimizing the Risk of Legionellosis Associated with Building Water Systems.

[25] World Health Organization (WHO) (2011). Guidelines for Drinking-Water Quality.

[26] Wang H., Bédard E., Prévost M., Camper A.K., Hill V.R., Pruden A. (2017). Methodological approaches for monitoring opportunistic pathogens in premise plumbing: A review. Water Res..

[27] Edagawa A., Kimura A., Doi H., Tanaka H., Tomioka K., Sakabe K., Nakajima C., Suzuki Y. (2008). Detection of culturable and nonculturable Legionella species from hot water systems of public buildings in Japan. J. Appl. Microbiol..

[28] Stout J.E., Yu V.L., Yee Y.C., Vaccarello S., Diven W., Lee T.C. (1992). Legionella pneumophila in residential water supplies: Environmental surveillance with clinical assessment for Legionnaires’ disease. Epidemiol. Infect..

[29] Leoni E., De Luca G., Legnani P.P., Sacchetti R., Stampi S., Zanetti F. (2005). Legionella waterline colonization: Detection of Legionella species in domestic, hotel and hospital hot water systems. J. Appl. Microbiol..

[30] Kehres D.G., Maguire M.E. (2003). Emerging themes in manganese transport, biochemistry and pathogenesis in bacteria. Fems Microbiol. Rev..

[31] Rhoads W.J., Pruden A., Edwards M.A. (2016). Convective Mixing in Distal Pipes Exacerbates Legionella pneumophila Growth in Hot Water Plumbing. Pathogens.

[32] Serrano-Suarez A., Dellunde J., Salvado H., Cervero-Arago S., Mendez J., Canals O., Blanco S., Arcas A., Araujo R. (2013). Microbial and physicochemical parameters associated with Legionella contamination in hot water recirculation systems. Environ. Sci. Pollut. Res..

[33] Halabi M., Wiesholzer-Pittl M., Schoberl J., Mittermayer H. (2001). Non-touch fittings in hospitals: A possible source of *Pseudomonas aeruginosa* and *Legionella* spp.. J. Hosp. Infect..

[34] Hosein I.K., Hill D.W., Tan T.Y., Butchart E.G., Wilson K., Finlay G., Burge S., Ribeiro C.D. (2005). Point-of-care controls for nosocomial legionellosis combined with chlorine dioxide potable water decontamination: A two-year survey at a Welsh teaching hospital. J. Hosp. Infect..

[35] Kusnetsov J., Torvinen E., Perola O., Nousiainen T., Katila M.L. (2003). Colonization of hospital water systems by legionellae, mycobacteria and other heterotrophic bacteria potentially hazardous to risk group patients. APMIS Acta Pathol. Microbiol. Immunol. Scand..

[36] Allegheny County Health Department (1997). Approaches to Prevention and Control of Legionella Infection in Allegheny County Health Care Facilities.

[37] Clancy C.M., Department of Veterans Affairs; Veterans Health Administration (2014). VHA Directive 1061: Prevention of Healthcare-Associated Legionella Disease and Scald Injury from Potable Water Distribution Systems.

[38] Vickers R.M., Brown A., Garrity G.M. (1981). Dye-containing buffered charcoal yeast extract medium for the differentiation of members of the family Legionellaceae. J. Clin. Microbiol..

[39] ISO (1998). Water Quality—Detection and Enumeration of *Legionella*. International Organization for Standardization.

[40] ISO (2004). 11731-2:2004 Water Quality Detection and Enumeration of Legionella Part 2: Direct Membrane Filtration Method for Waters with Low Bacterial Counts.

[41] Eaton E., Rice E., Baird R. (2005). Standard Methods for the Examination of Water and Waste Water.

